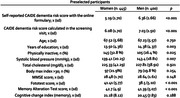# Gender differences in dementia risk factors and cognitive performance in the recruitment process of a FINGER‐like multidomain trial to prevent cognitive decline, the CITA GO‐ON trial

**DOI:** 10.1002/alz.088141

**Published:** 2025-01-09

**Authors:** Mikel Tainta, Myriam Barandiaran, Mirian Ecay, Ane Iriondo, Ainara Estanga, Miren Altuna, Carolina Lopez, Jon Saldias, Maite Garcia‐Sebastian, Cañada Marta, Maria de Arriba, Imanol Reparaz‐Escudero, Naia Ros, Jara Domper, Maider Mateo‐Abad, Pablo Martinez‐Lage

**Affiliations:** ^1^ Fundación CITA‐Alzhéimer Fundazioa, Centro de Investigación y Terapias Avanzadas ‐ Osakidetza, Organización Sanitaria Integrada Debabarrena (OSI) ‐ University of Deusto, San Sebastián, Guipúzcoa Spain; ^2^ CITA Alzheimer Foundation, Donostia‐San Sebastian Spain; ^3^ Biogipuzkoa Health Research Institute, Donostia‐San Sebastian Spain; ^4^ CITA Alzheimer Foundation, San Sebastian Spain; ^5^ Fundación CITA‐Alzhéimer Fundazioa, Centro de Investigación y Terapias Avanzadas;, San Sebastian, Guipúzcoa Spain; ^6^ Navarrabiomed, Hospital Universitario de Navarra (HUN)‐Universidad Pública de Navarra (UPNA), IdiSNA, Pamplona, Navarra Spain; ^7^ University of the Basque Country UPV/EHU, Donostia‐San Sebastian Spain; ^8^ BCC innovation ‐ Basque Culinary Center, Donostia Spain; ^9^ Biodonostia Health Research Institute, Donostia‐San Sebastian Spain; ^10^ Fundación CITA‐Alzhéimer Fundazioa, San Sebastian Spain

## Abstract

**Background:**

Sex and gender affect Alzheimer’s and Dementia differently. Some studies show that women are diagnosed at a later stage. This represents an essential challenge for healthcare and makes research challenging as gender biases may impact participant selection for clinical trials. We examined recruitment for the CITA GO‐ON trial, a lifestyle intervention trial to prevent cognitive decline.

**Method:**

The CITA GO‐ON study (ClinicalTrials.gov, NCT04840030) is a population‐based randomized clinical trial that aims to evaluate the effectiveness of a multidomain lifestyle intervention in preventing cognitive decline. Participants are older adults (60‐85 years) at high risk of dementia (CAIDE dementia risk score ≥ 6) using the Cognitive Change Index and Fototest and Memory Alteration Test (T@M) screening tools. Interested individuals fill out a REDCap online form that allows pre‐selection (by estimated CAIDE) for an individualized face‐to‐face screening visit to confirm eligibility. The descriptive analysis of the recruited sample and the comparison of dementia risk factors and cognitive tool performance between genders are presented.

**Result:**

Over 2500 subjects showed interest in the study by January 2022 through an online pre‐screening questionnaire. Of these, 60.4% were women (mean age, 67.52 years). After completing the online pre‐selection phase and the screening visit (n = 845), the representation of women dropped to 52.7% and 50.5%, respectively. Table 1 shows some preliminary data on the current recruitment process, which will be finished by Q2 2024. Preselected women have a lower mean CAIDE dementia risk score than men (p<0.001). Women also have fewer years of education and are less physically active. Blood pressure is higher in men. Although both sexes similarly report memory complaints, women perform better in cognition screening tools.

**Conclusion:**

Women seem to be more interested in initiatives promoting healthy aging. We have observed differences in dementia risk factors between men and women that could be related to sociocultural gender differences in this range of age. As previous studies have shown, in our study women perform better on brief cognitive tests, making them less likely to be selected. This highlights the importance of considering sex and gender for future analysis, trial designs, and recruitment strategies.